# Improving the Surface-Enhanced Raman Scattering Performance of Silver Nanodendritic Substrates with Sprayed-On Graphene-Based Coatings

**DOI:** 10.3390/s18103404

**Published:** 2018-10-11

**Authors:** Aida Mohammadi, Danielle Lilly Nicholls, Aristides Docoslis

**Affiliations:** Department of Chemical Engineering, Queen’s University, Kingston, ON K7L3N6, Canada; 16am65@queensu.ca (A.M.); 14dln3@queensu.ca (D.L.N.)

**Keywords:** surface-enhanced Raman scattering, silver dendrites, electric field-guided assembly, graphene-based coatings, sprayed coatings, illicit drug detection

## Abstract

This study examines the improvements in surface-enhanced Raman scattering (SERS) performance achieved when silver nanodendritic structures are coated with various graphene-based materials, namely graphene oxide (GO), reduced graphene oxide (rGO), and graphene nanoplatelets (GNPs). The tests are performed on our unique SERS-active substrates, prepared on the surface of planar microelectrode chips using an electric field-guided Ag nanoparticle assembly process. The graphene-based materials are introduced into the substrate by means of an in-house spray-coating technique. The SERS enhancement effect of each coating is examined as a function of spray nozzle passes (N) and optimal values are identified for each coating type. The enhancements found for GO, rGO, and GNP (6–9 graphene layers thick) coatings are 2.3 (N = 25), 2.5 (N = 5), and 1.6 (N = 1), respectively. Additionally, in comparison with their uncoated counterparts, substrates coated with rGO (N = 5) are shown to enhance the intensity of the methamphetamine (5 ppb) spectrum in artificial saliva by approximately 3-fold. Overall, it can be concluded that the introduction of GO or rGO to the SERS substrate using spray-coating, a simple and also scalable method, can produce substantial SERS performance enhancement.

## 1. Introduction

Surface enhanced Raman scattering (SERS) is an ultra-sensitive, non-destructive, and label-free spectroscopic technique, which is used for molecular sensing and chemical detection in a wide range of fields, including toxicology, environmental monitoring, and food safety [1,2,3,4]. Owing to its features, including small sample volume requirements, high selectivity, and a tremendous vibrational signal intensity enhancement (theoretically greater than 10^10^-fold) compared to spontaneous Raman [1], SERS has the potential to become a powerful point-of-need diagnostic technique that can “sniff out” trace quantities of chemical contaminants, food adulterants, illicit drugs [5,6] or explosives [7,8].

SERS is, primarily, of electromagnetic nature and originates from localized surface plasmon resonance (LSPR) at the surface of noble metallic nanostructures, of which materials comprising of silver are among the most effective in terms of plasmonic response [9]. However, one main drawback of conventional silver-based SERS substrates is their poor physical stability due to the oxidation that strongly impacts their sensitivity and performance consistency. To circumvent this limitation, SERS-active metallic nanostructures are usually covered by a stable protective layer or shell made from inert materials, such as metal oxides and carbon materials [10,11]. For example, Ju et al. reported silver nanoparticle substrates protected by small nitrogen-doped Graphene Quantum Dots (Ag NP@N-GQD) preserved SERS performance in a normal indoor environment for at least 30 days in both wet and dry states, in contrast to only 10 days for pure silver nanoparticles [12]. Among them, graphene and graphene-based derivatives, such as graphene oxide (GO) and reduced graphene oxide (rGO), are becoming favorable since their presence has also been associated with stronger SERS effects. The observed SERS signal enhancement is attributed to the contribution of a chemical enhancement mechanism originating from charge transfer effects between the graphene layer and the adsorbed molecules [9]. Moreover, graphene and its derivatives also combine a host of fascinating properties, such as high carrier mobility, great optical transparency, chemical inertness, and biological compatibility [11].

Improvements in SERS performance with the incorporation of graphene or its derivatives in SERS-active substrates have been demonstrated with a variety of methods, such as coating, simultaneous assembly of graphene and nanoparticles, or deposition of noble metal nanostructures on top of a pre-deposited graphene layer. Rhodamine 6G (R6G) detection at concentrations as low as 10^−14^ M was achieved for graphene-coated Ag “nanoflowers” supported on a Poly (methyl methacrylate) (PMMA) substrate [1]. Structures featuring a monolayer of graphene sandwiched between beam lithography-fabricated Ag nanostars and Au nanoparticles enabled sub-picomolar analyte detection capabilities [9]. It was shown that the simultaneous assembly of GO nanoplatelets with shape-controlled Ag NPs (octahedra) produced hybrid materials that improved the SERS signal up to 3-fold [13]. A nanoparticle-film gap (NFG) system, produced with graphene acting as sub-nanospacer between an Ag film and Ag nanoparticles, was shown to offer one of the highest SERS enhancement ratios reported to date for graphene-metal systems (1700-fold), which was nearly 115 times and 14 times larger than that of graphene on Ag film and graphene on Ag nanoparticles, respectively [14].

SERS-active substrates comprising noble metal dendritic nanoparticle structures constitute a new and promising class, with much superior performance compared to many other nanoparticulate substrates [15,16,17]. However, dendritic nanoparticle structures coated with graphene-based materials have rarely been studied. The coating of Ag dendritic and Ag nanoparticulate SERS substrates reported in the literature is accomplished through galvanic replacement reactions, chemical vapour deposition (CVD), or spin coating methods [18,19,20]. In one recent study, rGO was incorporated in Ag dendritic structures during the galvanic replacement between Ag+ ions and aluminum foil, resulting in SERS-active substrates with an enhancement factor of 5 × 10^4^ for Rhodamine B (RhB) and overall high spatial uniformity [18]. RhB signal enhancements up to 2.0-fold were reported due to the incorporation of rGO. The authors explained that the rGO sheets could efficiently adsorb more molecules, as well as facilitate the interaction between silver and RhB. In another study, GO-coated Ag nanodendrites produced through the galvanic replacement reaction of Ag nitrate solution on copper foils produced SERS-active substrates with remarkable sensitivity and stability [19]. The GO coating resulted in 2.4- to 2.7-fold enhancement in the spectrum of Rhodamine 6G (R6G) and allowed detection of R6G down to 10–11 M. However, the production of uniform graphene-based coatings over large surface areas still remains a big challenge.

We have recently demonstrated a facile, rapid and inexpensive method for the in situ preparation of “SERS-on-a-chip” substrates that enable ultrasensitive quantitative detection of small molecules. The method uses energized planar microfabricated electrodes deposited on an oxidized silicon wafer, and the spatially non-uniform AC electric field generated by them, to drive the assembly of colloidal Ag nanoparticles into dendritic structures that exhibit strong LSPR coupling. The functionality and sensitivity of the resulting SERS substrates have been demonstrated with the detection of small quantities of illicit drugs (cocaine) in oral fluids, food adulterants (melamine) in baby formula, pesticides in apple juice, explosives (DNT), etc. [21,22,23]. However, a strategy for further improving the SERS activity through the preparation of a two-layer structure, that combines the strong plasmonic effect of silver nanodendrites with graphene-based coatings, has not previously been explored by our group. 

To the best of our knowledge, this is the first study where the effects of three major graphene-based materials are examined side-by-side in terms of SERS performance enhancement. Moreover, this is also the first study where graphene-based coatings are applied to our recently reported Ag nanoparticle dendrites and compared with their uncoated counterparts. The graphene-based materials were introduced into the substrate by means of an in-house developed spray-coating technique. It is worth mentioning that the use of a spray-nozzle towards the functionalization (coating) of SERS substrates has not been studied previously. The SERS enhancement effect of each coating was examined as a function of spray nozzle passes (N) and optimal values were identified for each coating. The spraying method offers the advantages of allowing some control over solvent evaporation, nanoparticle aggregation prevention in the coating, and production of films with uniform thickness [24]. The spray-coating method is also amenable for industrial scale-up since the area to be coated can, in principle, be increased by sweeping the spray nozzle across a greater substrate area. 

## 2. Materials and Methods

### 2.1. Silver Nanoparticles

**Figure 1:**
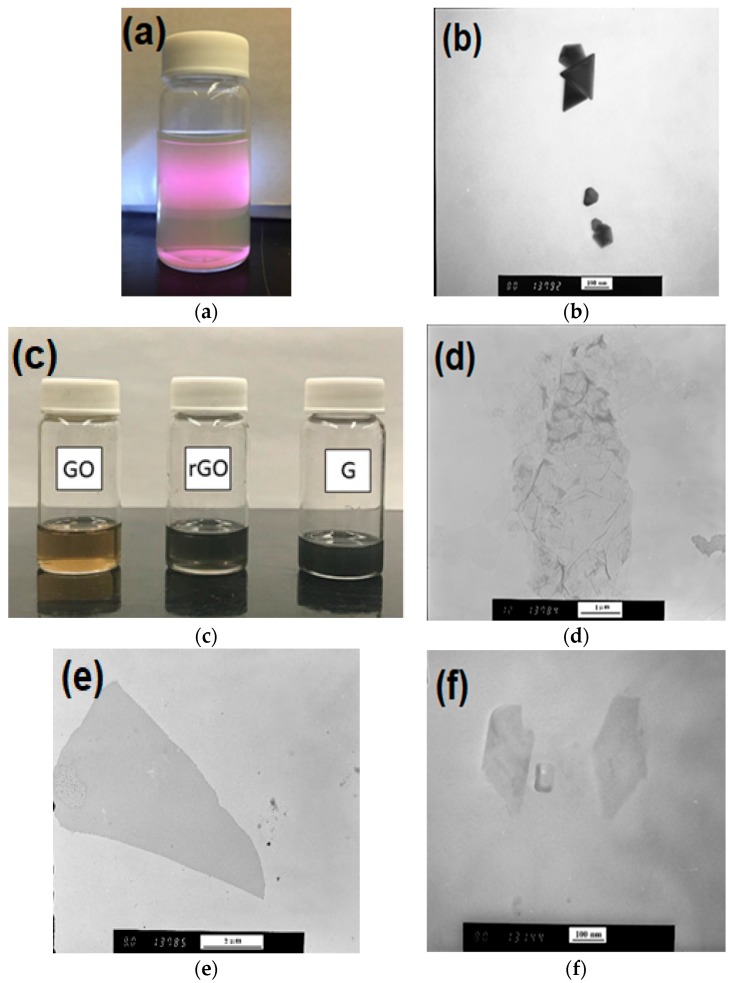
(**a**) Image of the synthesized colloidal silver nanoparticle suspension used for assembling the dendrites. (**b**) TEM image of the corresponding silver nanoparticles (Scale bar: 100 nm). (**c**) Suspensions (0.1 mg/mL) of the graphene-based materials used in this study and corresponding TEM images: (**d**) graphene oxide (GO) dispersed in ethanol (scale bar: 1 μm); (**e**) reduced graphene oxide (rGO) dispersed in N-Methyl-2-pyrrolidone (NMP) (scale bar: 2 μm); (**f**) graphene nanoplatelets (GNPs) dispersed in NMP (scale bar: 100 nm).

The silver nanoparticles were prepared by a seedless photo-assisted citrate reduction method under the irradiation of blue light-emitting diodes (LEDs) according to a previously reported method [25]. Silver nitrate (AgNO_3_) and sodium citrate (C_6_H_5_Na_3_O_7_) with a normal purity of 99% were obtained from Sigma-Aldrich, Oakville, ON, Canada. Millipore^®^ water (18.2 MΩ·cm) was used in all experiments. In brief, 1 mL of sodium citrate (4.5 × 10^−1^ M) and 1 mL of silver nitrate (1.0 × 10^−2^ M) were mixed with 98.0 mL of pure water. The mixture was subsequently irradiated with 30 blue LEDs (Inspired LED lightening bars and strips blue with λ_max_ = 465 nm and average power = 240 mW, Mouser Electronics Inc., Kitchener, ON, Canana) at room temperature. Silver colloids containing a mixture of decahedral nanoparticles (app. 60% (Mouser electronics, Kitchener, ON, Canada), as determined by Transmission Electron Microscopy, TEM) and nanoparticles of triangular shape (Figure 1a,b), were obtained after 24 h of irradiation. The silver nanoparticle suspension was concentrated 10 times by centrifugation at 1800× *g* for 30 min prior to use. 

### 2.2. Graphene Oxide Dispersion

GO was obtained from Graphenea Inc. (Saint Sebastian, Spain) in the form of nanoplatelets dispersed in water (4 mg/mL). The dispersion was centrifuged at 1800× *g* for 10 min and the precipitate was re-suspended in ethyl alcohol to the concentration of 0.1 mg/mL. The new dispersion (see Figure 1c) was subjected to 30 min of ultrasonication in a 140 W ultrasonic bath (Aquasonic 50D, VWR Scientific, New York, NY, USA) prior to use in the coating process. A TEM image of GO is shown in Figure 1d. 

### 2.3. Reduced Graphene Oxide 

The rGO was prepared through the chemical reduction of GO using benzylamine (99%, Sigma-Aldrich) as a reducing and stabilizing agent [26]. In a typical synthesis, 2 mL of GO aqueous dispersion with concentration of 0.5 mg/mL was added into 4 mL of deionized water (H_2_O), followed by addition of 200 μL of benzylamine. After stirring for 30 min at room temperature, the mixture was heated to 90 °C over a 120 min period and a black rGO colloidal dispersion was achieved. The excess benzylamine was removed by centrifuging twice at 1800× *g* for 30 min. The precipitate (rGO) was dried overnight in a vacuum oven (0.07 mPa) at 65 °C and then re-dispersed in 10 mL N-Methyl-2-pyrrolidone (NMP, 99.5%, Sigma-Aldrich, Oakville, ON, Canana) to a final concentration of 0.1 mg/mL, as depicted in Figure 1c. A TEM image of rGO is shown in Figure 1e. 

### 2.4. Graphene Nanoplates (GNPs)

GNPs were produced from graphite exfoliated using a Microson ultrasonic cell disruptor equipped with a 3 mm diameter sonicating tip. Natural graphite powder (purity 95% and 80 mesh) was supplied by Eagle Graphite and purified (99%) with anhydrous ethyl alcohol and acetone. Graphite powder (5 wt%) was added to Pluronic^®^ F127 (1 wt%, Sigma-Aldrich) as a surfactant solution and stirred until adequately mixed. The graphite-surfactant solution was placed in a temperature-controlled bath to maintain temperature at approximately 20 °C [27]. The solution was left to exfoliate for 4 h and then was centrifuged at 1800× *g* for 30 min. The supernatant was dried in a vacuum oven (0.07 mPa) at 65 °C and then re-dispersed in 10 mL NMP to a final concentration of 0.1 mg/mL, a depicted in Figure 1c. A TEM image of GNPs is shown in Figure 1f. 

### 2.5. Microchip Fabrication 

The microfabrication of electrodes was carried out at Nanofabrication Kingston (NFK, Innovation Park, Kingston, ON, Canada) via maskless photolithography on silicon wafers, followed by electron beam metal film evaporation and liftoff. In addition, the negative photoresist SU-8 (MicroChem Corp, Westborough, MA, USA) was used with the Intelligence Micro-Patterning (IMP) maskless photolithography system to allocate the microelectrode pattern to the silicon substrate. A 5 nm layer of chrome was used to promote the adhesion of the deposited Au layer (100 nm thickness) to the silicon substrate.

### 2.6. SERS Substrate Preparation

A total of 5 μL of silver nanoparticle dispersion in water was placed over the individual microelectrode chips center using a micropipette. The collection was run for 9 min at AC (alternating current) frequency of 10 Hz and voltage of 3 Vpp (peak-to-peak) by simultaneously applying a positive DC (direct current) bias of 0.5 Vpp [22]. Following nanoparticle deposition, the chip was washed with water and dried in a stream of air. Subsequently, the prepared SERS substrates with silver dendritic structures were placed in a vacuum oven (DZF-6020-UL, MTI Corporation, Richmond, CA, USA) at 0.07 MP and 80 °C for 1 h.

### 2.7. Sprayed Graphene-Based Coatings 

The different synthesized graphene-based colloidal dispersions passed through a gravity-fed airbrush (VL-SET, Paasche Airbrush Company, Chicago, IL, USA) at 30 psi, for which nitrogen was the gas-phase carrier. The SERS substrates prepared in the first procedure were sprayed at a nozzle-to-surface distance of 30 cm and coated by a different number of horizontal passes (1, 5, 10, 15, 20, and 25) allowing for accurate control over the density of the GO, rGO, and GNP depositions. GO-based coatings were applied at 25 °C, whereas the rGO- and GNP-based ones were applied at 80 °C using a hot plate, in order to approximately equate the solvent evaporation time in all cases. Once coated, the silver dendritic SERS substrates were placed to dry in a vacuum oven (0.07 MP) at 80 °C for 2 h.

### 2.8. Characterization 

Transmission electron microscopy (TEM) was performed at the Electron Microscopy Facility (Botterell Hall, Queen’s University) using a Hitachi H-7000. Scanning electron microscopy (SEM) and energy dispersive X-ray spectroscopy (EDX) was performed at the Queen’s Facility for Isotope Research, on a MLA 650 Field Emission Gun (FEG) environmental SEM (Thermo Fischer Scientific, Hillsboro, OR, USA), at a voltage of 5.00 kV. For measuring the Raman signals of dye molecules, a 5 μL droplet was dispersed carefully with a micropipette on the surface of the as-prepared SERS substrates and left to dry. Substrates containing spreading droplets (contact angle ~0°) were not included in the measurements since they resulted in a significantly lower surface concentration of analyte. A HORIBA/Jobin-Yvon (Edison, NJ, USA) Raman Spectrometer (Model: LabRAM) with a 632.8 nm He/Ne laser (17 mW), 1800 l/mm grating and an Olympus BX-41 microscope system were used. The collection of spectra was performed in the backscattered mode under the following conditions: ×100 microscope objective, 500 μm pinhole, 500 μm slit width, laser filter 10×, for a sampling time of 5 s with 4 repeats. All Raman spectra were background corrected through polynomial subtraction, and noise was reduced with a Savitsky-Golay filter. 

## 3. Results

### 3.1. Electric Field-Assisted Assembly of Dendritic Ag Nanoparticle Structures

**Figure 2:**
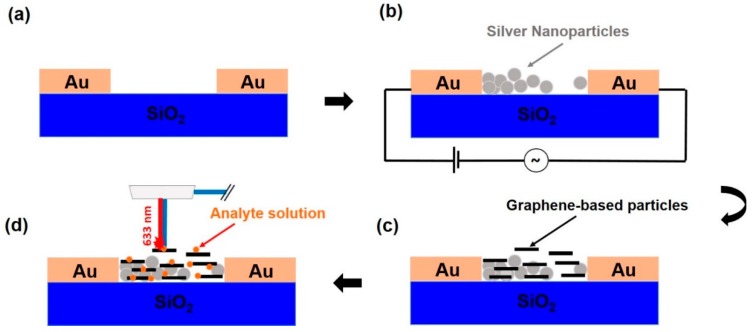
Schematic presentation of G-surface-enhanced Raman scattering (SERS) substrate preparation and detection process. (**a**) The individual electrode pairs are activated by an electric signal. (**b**) This procedure deposits silver nanoparticles into a SERS-active dendritic structure. (**c**) Deposition of a graphene-based coating on the SERS-active substrate through spray-coating. (**d**) Drop-casting of analyte (orange dots) onto the G-SERS substrate for Raman testing.

The steps involved in the preparation of the coated SERS-active substrates (“G-SERS substrates”) are illustrated schematically in Figure 2. Figure 2a is a schematic of the cross section of the planar microelectrode structure used in the experiments. As we have shown in a previous publication [20], the spatially non-uniform electric field that is created when an AC signal is applied across these microelectrodes can drive the assembly of colloidal silver into dendritic, SERS-active nanoparticle structures on the microelectrode surface (Figure 2b). Subsequently, the dendritic structures can be functionalized further by spray-coating them with graphene-based materials (Figure 2c). Performance testing of the SERS activity of the various coatings thus produced was performed with Raman micro-spectroscopy by the drop-casting R6G on the substrate’s surface (Figure 2d).

**Figure 3:**
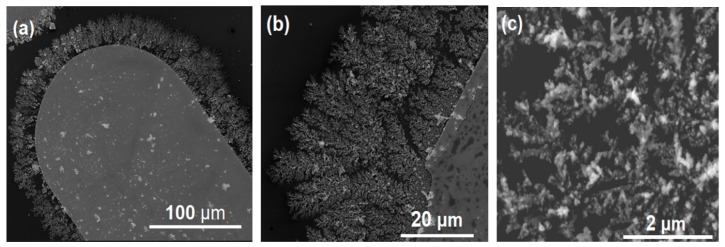
SEM images of dendritic silver nanoparticles structures at different magnifications. (**a**) Image of formed structures around the gold microelectrode. (**b**) Detail of the structure at the microelectrode edge. (**c**) Close-up showing individual nanoparticles participating in the structure. Nanoparticle assembly conditions, 10 Hz, 3 Vpp with 0.5 V DC bias.

The morphology of the Ag nanodendritic SERS-active structures formed around the gold planar microelectrodes can be observed in the SEM images shown in Figure 3, where details of these structures are revealed at various magnifications. As can be seen, the structures are continuous and comprise numerous extended fractal nanoparticle branches. The high density of nanoparticle contacts formed within the clusters, as well as the multiple contacts between the edges of neighboring branches and junctions of adjacent branches results in high surface density of “hot spots” and, subsequently, high SERS activity [18]. Moreover, this type of dendritic nanostructure uses nanoparticles as building blocks and therefore it should be clearly differentiated from the electrochemically grown dendrites reported earlier by others [18,19]. An additional evidence to this is the fact that dendritic structure formation only occurs under low AC field frequencies (<50 Hz) but not under a DC field, as in the case of previous studies.

It is interesting to note that the structures are seen to form along the complete microelectrode edge and not only in the narrowest gap between microelectrodes, where the electric field gradient is the strongest. Therefore, it can be concluded that the electric field intensity, which correlates with the rate of nanoparticle transport to the assembly sites (dielectrophoresis), is sufficiently high everywhere. On the other hand, the fractal geometry of the formed nanoparticle structures suggests that the assembly probably takes place under a mass transfer-controlled regime due to strong short-range particle-particle interactions. The exact mechanism(s) that governs the formation of these dendritic nanoparticle structures is currently under investigation by our group.

### 3.2. Dendrites Coated with Graphene-Based Derivatives.

Three different G-SERS substrates were produced by spray-coating the Ag dendrites with either of the following three graphene-based derivatives (see Figure 1c): graphene oxide (GO), reduced graphene oxide (rGO), and graphene nanoplatelets (GNPs). The GO has lateral dimensions on the order of 1 μm (see Figure 1d) and forms single-layer platelets when cast from suspension in ethanol [28]. As shown in Figure 1e, the rGO, which was produced from the reduction of the aforementioned GO, also maintains very similar physical dimensions. One the other hand, the GNPs (see Figure 1f), which were prepared in-house via surfactant-assisted ultrasonication of graphite in water, were found to have much smaller lateral dimensions (200–300 nm) and estimated aspect ratios in the range 70–100, i.e., a thickness of about 6–9 graphene layers. Details on the morphological and physical characterizations performed on the GNPs can be found elsewhere [27]. 

Considering the uneven surface topography of the Ag dendrites, it can easily be understood that accurate thickness measurement of the formed coatings is not possible. For this reason, the coatings’ thickness is only reported in terms of the number of spray nozzle passes (N) over the substrate. As a point of reference, our previous work showed that 15 nozzle passes produce a complete monolayer (95%–98% coverage) of GO on a flat silicon dioxide surface [27]. In the present study N was varied between 0 and 25; therefore, it is assumed that in all cases the mean coating thickness should be between 0 and 2 platelet layers thick.

**Figure 4:**
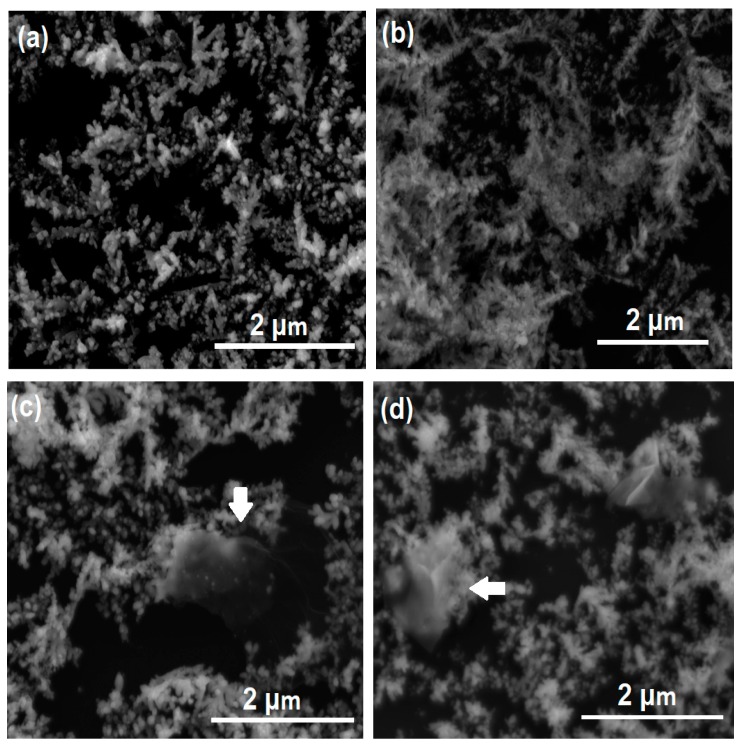
Typical SEM images of different G-SERS substrates. (**a**) Uncoated silver dendrites (N = 0). (**b**) GO-coated dendrites (N = 25). (**c**) rGO-coated dendrites (N = 5). (**d**) GNP-coated dendrites (N = 1). The white arrows indicate visible graphene-based coatings on the dendrites.

Figure 4 shows characteristic SEM images of the dendritic substrates after spray coating. The first observation that can be made is that the dendritic structures remain undisturbed after the coating process, which is evidence of the robustness of these substrates. Although the coatings cannot be resolved under SEM, some of the deposited material (rGO platelets or GNP) is visible, as indicated by the arrows in Figure 4c,d, respectively. Additionally, indirect evidence of the presence of the coatings is the lack of clarity (haziness) of the images in Figure 4b,c (GO and rGO coatings, respectively), which is attributed to electron charging effects due to the poor electrical conductivity of these coatings. In contrast, the clarity of Figure 4a (uncoated dendrites) is much higher. The latter can also be said for Figure 4d, primarily due to the GNP surface density being much lower (N = 1). As explained later, the number of passes for each coating shown in Figure 4 correspond to the optimal number in terms of SERS activity.

**Figure 5:**
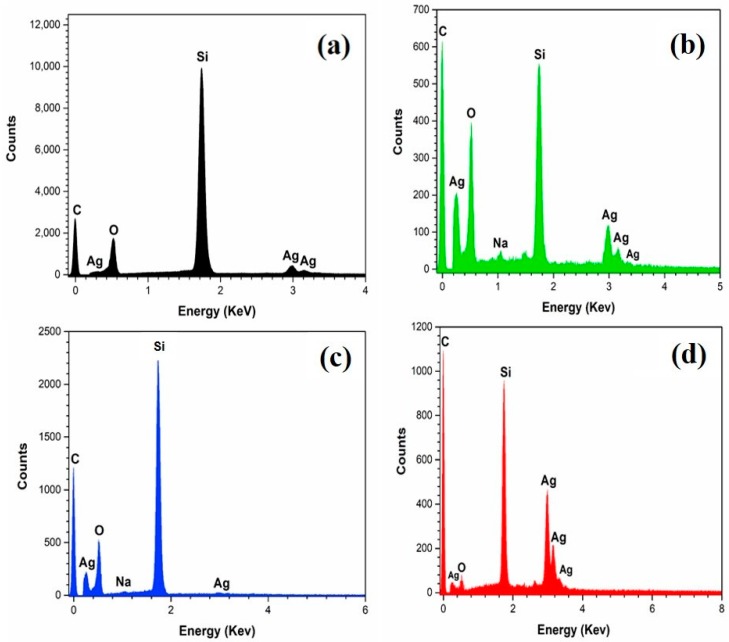
EDX spectra of G-SERS substrates. (**a**) Uncoated dendrites (N = 0). (**b**) GO-coated dendrites (N = 25). (**c**) rGO-coated dendrites (N = 5). (**d**) GNPs-coated dendrites (N = 1). N indicates the number of spray nozzle passes over the dendritic substrate.

Evidence for the presence of coatings was also provided by the EDX (Energy-dispersive X-ray spectroscopy [29] scans, as shown in Figure 5. Figure 5a is the EDX scan from an uncoated dendritic substrate. The carbon peak shown in Figure 5a is coming from carbon contained in sodium citrate (C_6_H_5_O_7_Na_3_), the surfactant used for the stabilization of silver nanoparticle in suspension. Figure 5b represents a sample of GO-coated dendrites (N = 25). The low C ÷ O intensity ratio (app. 1.5) is very characteristic for the case of GO. Additionally, the very strong carbon peak with respect to the Ag peaks is an indication of very good surface coverage. For the case of rGO coating (Figure 5c), the Si ÷ C peak ratio is higher, which indicates a lower surface coverage compared with the case of GO. This is expected as N = 5 in this case. Moreover, the C ÷ O intensity ratio is also higher than before (app. 2.0) as a result of the GO reduction process. Considering that a good amount of the oxygen present in the system comes from the SiO_2_ background (see Figure 1a), we can say that the reduction of the GO platelets was very effective. Finally, Figure 5d (N = 1) shows that the C ÷ O intensity ratio is extremely high, i.e., the GNPs were not oxidized during the exfoliation and purification steps. The relatively strong intensity of carbon (high C ÷ Si ratio) in Figure 5d (N = 1) resulted from an EDX spectrum acquired deliberately from an area where SEM showed the presence of GNPs (see Figure S1, Supplementary Information). EDX scans from randomly selected location in the GNP coating (N = 1) typically produced spectra similar to that shown in Figure 5a (control). Overall, these results are in qualitative agreement with our expectations as they prove the presence of a significant amount of carbon on the surface of the dendrites. 

### 3.3. SERS Performance Assessment 

**Figure 6:**
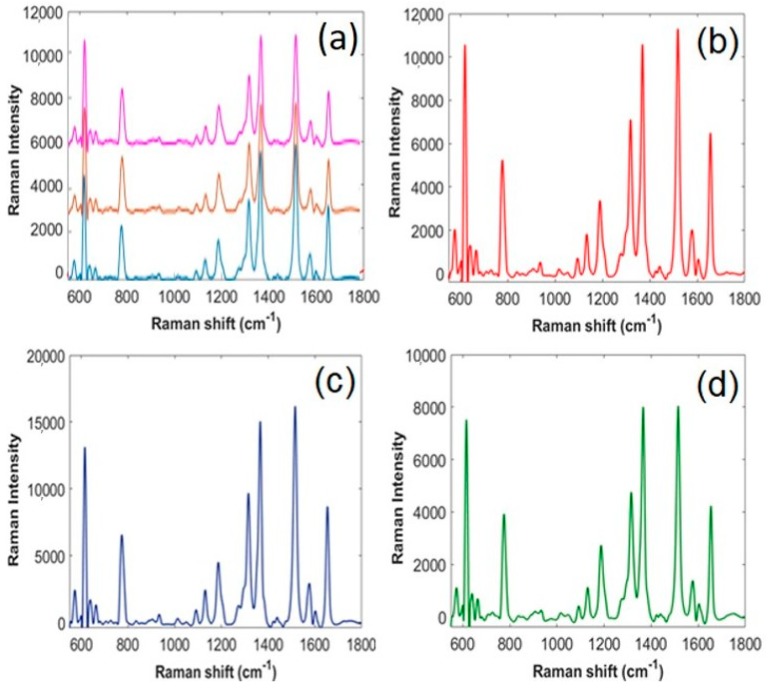
SERS spectra of 10^−5^ M R6G on different G-SERS substrates. (**a**) Uncoated dendritic substrate. (**b**) GO-coated dendrites (N = 25). (**c**) rGO-coated dendrites (N = 5). (**d**) GNP-coated dendrites (N = 1).

R6G, a Raman-active cationic dye, was used to assess the SERS activity of dendritic substrates, on which graphene-based coatings were applied. Figure 6a is a cluster of SERS spectra collected at various, randomly selected, locations on the uncoated dendritic substrate. The enhancement factor of these dendritic substrates was assessed in a previous study and found to be approximately 4 × 10^5^ [22]. As can be seen, the intensity variation among these spectra is small, indicating that the SERS performance of the substrate is spatially consistent. The peak at 618 cm^−1^ allocated to C–C–C bond stretching vibration, the peaks at 1316, 1368, 1516 and the peak of 1655 cm^−1^ assigned to C–C stretching modes, whereas the peak at 776 cm^−1^ is ascribed to the out-of-plane vibration of deformed C–H bonds [30]. From the spectral assignments, one can see that the strong enhancement seems to be associated with aromatic C–C–C bonds in-plane vibration of the R6G molecular, suggesting that the aromatic rings in R6G are closely interacting with the prepared SERS substrates. The large Raman enhancement of the peak in the 616–618 cm^−1^ range is due to π–π stacking and the lone pair of electrons in the oxygen-containing groups between the R6G molecule and the substrate. Interestingly, the peak at 1580 cm^−1^ assigned to C–O–C bond stretching vibration of R6G molecule overlays with the G band of graphene-based materials (Figure S2, Supplementary information). It is reported that for the comparatively high concentrations, this peak intensity is equal to the sum of the 1580 cm^−1^ peak of R6G and G band of graphene, while for the relatively low concentrations, this peak is mainly equal to the G band of graphene [1].

Figure 6b–d shows R6G spectra collected under the same conditions from the surface of GO- (N = 25), rGO- (N = 5) and GNP- (N = 1) coated dendrites. Examined side by side, these spectra show that the intensity of the SERS spectra of R6G can improve significantly with the incorporation of a graphene-based layer. Whereas the SERS effect produced by the Ag dendrites is purely due to the effect of the electromagnetic mechanism, the addition of a graphene-based material also gives rise to SERS enhancement attributed to the chemical mechanism. An aromatic dye molecule, such R6G, lies approximately parallel to the surface of graphene, resulting in π–π interaction that lead to enhanced charge transfer (chemical mechanism) between them [11]. As seen here, and also confirmed by other reports in the literature, the final SERS enhancement is the result of the combination of the two aforementioned mechanisms [18,19].

The strong topographic irregularity exhibited by the Ag dendritic structures does not permit direct measurements of coating thickness and surface coverage. Therefore, the SERS enhancement produced by the coatings is examined here as a function of N over the substrate. Although not direct or precise, this approach still allows us to observe trends in the performance of the coatings and also identify the maximum possible effect that can be achieved in each case under the present experimental conditions. To accomplish this, an array of dendritic substrates was produced having as variables the type of spray-coated material (GO, rGO, GNP) and the number of spray nozzle passes used during the coating process (N = 1, 5, 10, 20, 25). The results are expressed in terms of the enhancement ratio, $R_{N}^{i}$, which compares the peak intensities in the SERS spectra of R6G produced by coated and uncoated substrates, using the equation:

(1) $$\;R_{N}^{i}=\frac{\;\mathrm{Peak}\;\mathrm{intensity}\;\mathrm{at}\;618\;{\mathrm{cm}}^{-1}\;\mathrm{from}\;\mathrm{coating}\;“i”\;\mathrm{after}\;N\;\mathrm{passes}}{\mathrm{Mean}\;\mathrm{peak}\;\mathrm{intensity}\;\mathrm{at}\;618\;{\mathrm{cm}}^{-1}\;\mathrm{from}\;\mathrm{uncoated}\;\mathrm{dendrites}}\;$$

A total of 21 SERS intensity measurements were initially performed for each N and coating type, i.e., 3 substrates × 7 measurements per substrate at pre-determined locations around the electrode edges. For the case of rGO, the number of measurements was later doubled, as explained below. Subsequently, the corresponding enhancement ratio for each measurement was produced by dividing the intensity at 618 cm^−1^ by the mean peak intensity of the uncoated dendritic substrates. 

**Figure 7:**
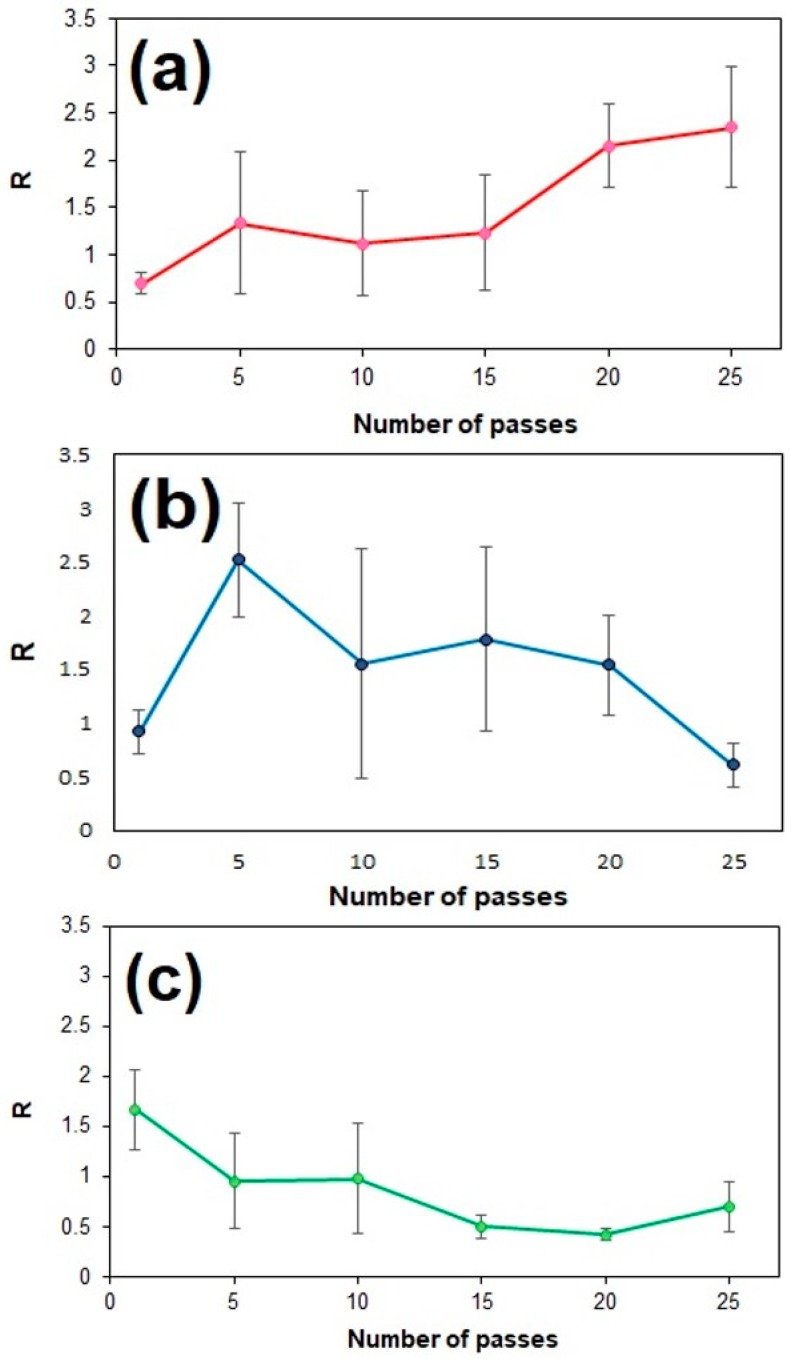
Enhancement ratio as a function of spray nozzle passes for different G-SERS substrates. (**a**) GO coated dendrites. (**b**) rGO-coated dendrites. (**c**) GNP-coated dendrites.

The results of this examination are summarized in Figure 7. Interestingly, different trends are seen for each type of coating. Figure 7a illustrates the performance of GO coatings with respect to the spray nozzle passes. As can be seen, one pass does not enhance the SERS performance of the substrate since the deposited GO is probably too little to make a significant contribution. To put that in context, our previous study showed that 15 spray nozzle passes are necessary for the formation of a complete monolayer of GO on a flat SiO_2_ substrate. However, by increasing the passes to N = 5, a 33% increase in the enhancement ratio is achieved. This positive trend continues all the way to the maximum number of passes that have been attempted (N = 25), for which $R_{25}^{GO}=2.3$. From these results it can be inferred that the GO platelets are distributed regularly over the Ag dendrite surface with every additional pass and intimate contact is achieved between them and the SERS hot spots of the substrate. Another contributing factor to the observed enhancement may be the high surface density of active oxygen sites presented by the GO, which render them an excellent adsorbent for the R6G molecules. A higher surface density of active oxygen sites can improve the graphene-metal/molecule binding, leading to an intensified Raman signal [31]. As the number of spray nozzle passes increases, the surface density of hot-spots connected with GO platelets also increases, resulting in an overall higher enhancement ratio. However, it is still currently unclear how much of the overall observed enhancement is attributable to the charge transfer mechanism. 

Figure 7b depicts the effect of N on the enhancement ratio produced by the rGO coatings. Same as before, for N = 1 rGO did not show any improvement in the enhancement ratio due to insufficient coverage. However, a dramatic increase in the value of the enhancement ratio is observed for N = 5, for which $R_{5}^{RGO}=2.5$. This performance is not only slightly better than what was observed earlier for GO, but also requires much fewer spray nozzle passes. It also seems to coincide with the point of peak performance for this type of coating. It must be mentioned here that the results of Figure 7b is the combination of two sets of measurements (2 × 21 points for each N). The repetition of the original data set was deemed necessary in order to confirm the existence of a local maximum for N = 5. Indeed, the presence of a local maximum at N = 5 was observed in both tests, with corresponding enhancement ratios (N = 5) equal to 2.9 and 2.1, respectively. For 5 < N < 25, the value of the enhancement ratio is lower but remains almost constant (around 1.5), which is in contrast to the behavior exhibited by GO. Finally, for N = 25, the SERS performance of the coating is poorer than that of the control. This is very likely an indication that the positive effects that the rGO platelets produced in terms of SERS enhancement now begin to be overshadowed by hot-spot masking effects. An explanation of the local maximum value that is exhibited by rGO around N = 5 is currently under investigation by our group.

Finally, the results from the GNP coatings are presented in Figure 7c. The trends here are again different than those seen before. Maximum performance enhancement is achieved only after the first pass ($R_{1}^{GNP}=1.6$), beyond which the system shows an almost monotonic decline in performance. It seems that the presence of some graphene on the surface of the dendrites produces favorable results; however, this benefit is quickly negated by the masking of hot-spot sites by the GNPs. An important reason for this behavior is the multilayered structure of GNPs, between 6–9 graphene layers [27], which has been shown to produce much weaker chemical enhancement [32]. 

Finally, Table 1 summarizes the best SERS performance ($R_{N}^{i}$) that was found for each one of the three coatings tested and the respective number of passes. Our previous study on sprayed GO film formation concluded that the coating thickness and consistency is strongly dependent on the selection of the dispersion medium used in the spraying process [28]. Ethanol was found to be the dispersion medium that produces the most uniform GO coating. However, in the present study, GNP and rGO were suspended in NMP, as it was shown to be among the most effective dispersion media for these materials [33]. Although optical and SEM images did not reveal any distortion of the dendritic structures after spraying, the role of the dispersion medium (solvent) on the final distribution and performance of the formed coatings should also be considered in future studies.

**Figure 8:**
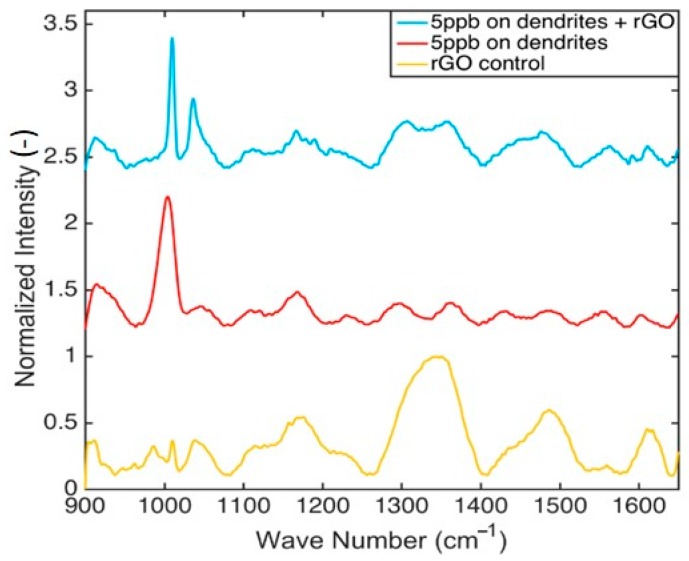
Normalized SERS spectra for methamphetamine (5 ppb) in artificial saliva: performance comparison between rGO-coated (blue line) vs. uncoated (red line) dendritic substrates. The spectrum of solvent on an rGO-coated substrate (yellow line) is included for reference.

The ability of the coated and uncoated substrates to detect illicit drugs was compared in tests using methamphetamine as the target analyte. Specifically, 5 μL of methamphetamine solution in artificial saliva were drop-cast on the surface of the substrates, left to dry and then rinsed with DI water prior to each measurement. The concentration of methamphetamine in the samples was 5 ppb, which is well below the legal limit of detection for a positive test (50 ppb) in Canada [34]. A total of 9 substrates (3 substrates per experimental condition) were used in the tests and 10 measurements were acquired at random locations on each substrate. Characteristic spectra for methamphetamine in artificial saliva on rGO-coated (N = 5) and uncoated substrates can be seen in Figure 8. The SERS spectrum of a blank sample (artificial saliva on rGO-coated substrate) is also included for reference. Analysis of the spectra revealed that the characteristic methamphetamine peak at 1038 cm^−1^ was intensified by an average of 3.9 times (S.D.: 3.5) on rGO-coated substrates, as compared to uncoated substrates. Moreover, the characteristic methamphetamine peak at 1010 cm^−1^ was sharper and intensified by an average of 2.1 times (S.D.: 1.4) on rGO-coated substrates. These results are in good agreement with our previous conclusions using R6G and suggest that a 2- to 3-fold SERS signal enhancement should be expected when an rGO-coating is applied. Although a definitive answer cannot presently be offered as to whether N = 5 also presents the optimum coating for Methamphetamine or other chemical analytes, a limited number of measurements performed on rGO coatings with N = 1 and N = 10 consistently gave weaker SERS signals for Methamphetamine than N = 5.

## 4. Conclusions

To the best of our knowledge, this is the first study where the effects of three major graphene-based materials were examined side-by-side in terms of SERS performance enhancement. Moreover, the study was performed on our unique and very promising, SERS-active substrates, namely colloidal Ag nanodendrites. The substrates were prepared on the surface of planar microelectrode chips using a, bottom up, electric field-guided Ag nanoparticle assembly process. The graphene-based materials were introduced into the substrate by means of an in-house developed spray-coating technique. The SERS enhancement effect of each coating was examined as a function of spray nozzle passes (N) and optimal values were identified for each coating. The enhancements that were found for GO, rGO, and GNP coatings are 2.3 (N = 25), 2.9 (N = 5), and 1.6 (N = 1), respectively. GO appeared to monotonically increase the SERS enhancement of the substrate with increasing N, up to the maximum value tried in this study (N = 25), at which the coating is assumed to be between 1–2 nanoplatetelet layers thick. rGO produced a better effect at only N = 5, beyond which the performance began to deteriorate. Although initially beneficial (N = 1), the performance of the GNP coating declined with more passes and finally became inferior to the control substrate. The GNPs used in this study comprised 6–9 graphene layers, which proves to be too thick for providing substantial effects. In tests involving methamphetamine (5 ppb) detection in artificial saliva, the rGO-coated substrates (N = 5) were found to generate a 2- to 3-fold stronger signal compared to uncoated dendrites. Overall, it was shown that substantial performance enhancement can be gained with the introduction of GO or rGO by using spray-coating, a method that is not only simple to perform, but also scalable. 

## Figures and Tables

**Table 1:** Optimal number of spay nozzle passes for each graphene-based coating studied.

Coating	Solvent	Optimal Passes, N	$R_{N}^{i}$	Standard Deviation (S.D.)
GO	Ethanol	25	2.3	0.64
rGO	NMP	5	2.5	0.53
GNP	NMP	1	1.6	0.45

Note: standard deviations for all experimental points can be found in Table S1 (Supplementary info).

## Supplementary Materials

The following are available online at http://www.mdpi.com/1424-8220/18/10/3404/s1, Figure S1: SEM image used in the acquisition of the EDX spectrum shown in Figure 5d. The circle indicates the location of the GNP(s); Figure S2: Raman spectra of graphene-coatings: (a and b) GO (N=25) on a flat substrate and Ag dendrites, respectively; (c and d) rGO (N=5) on a flat substrate and Ag dendrites, respectively; (e, f) GNP (N=1) on a flat substrate and Ag dendrites, respectively. A Au-coated (CVD) polished Si wafer was used as flat substrate; Table S1: Standard deviations and relative standard deviations (%) for the data displayed in Figure 7.
