# Supplementary material for: Improving the Surface-Enhanced Raman Scattering Performance of Silver Nanodendritic Substrates with Sprayed-On Graphene-Based Coatings
Source: Sensors (Basel). 2018 Oct 11;18(10):3404. doi: 10.3390/s18103404 (PMC6209902; doi:10.3390/s18103404)
Supplement: Supplementary file 1 [file sensors-18-03404-s001.pdf]

sensors

Supplementary Information

Improving the surface-enhanced Raman scattering performance of silver nanodendritic substrates with sprayed-on graphene-based coatings

Aida Mohammadi, Danielle Lilly Nicholls and Aristides Docoslis *

Department of Chemical Engineering, Queen's University, Kingston, ON, K7L 3N6 Canada; 16am65@queensu.ca (A.M.); 14dln3@queensu.ca (D.L.N)

* Correspondence: docoslis@queensu.ca; Tel.: +1-613-533-6949

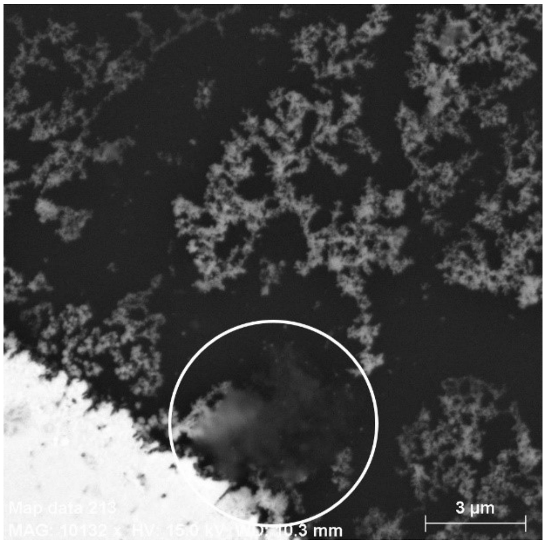

Figure S1. SEM image used in the acquisition of the EDX spectrum shown in Fig. 5d. The circle indicates the location of the GNP(s).

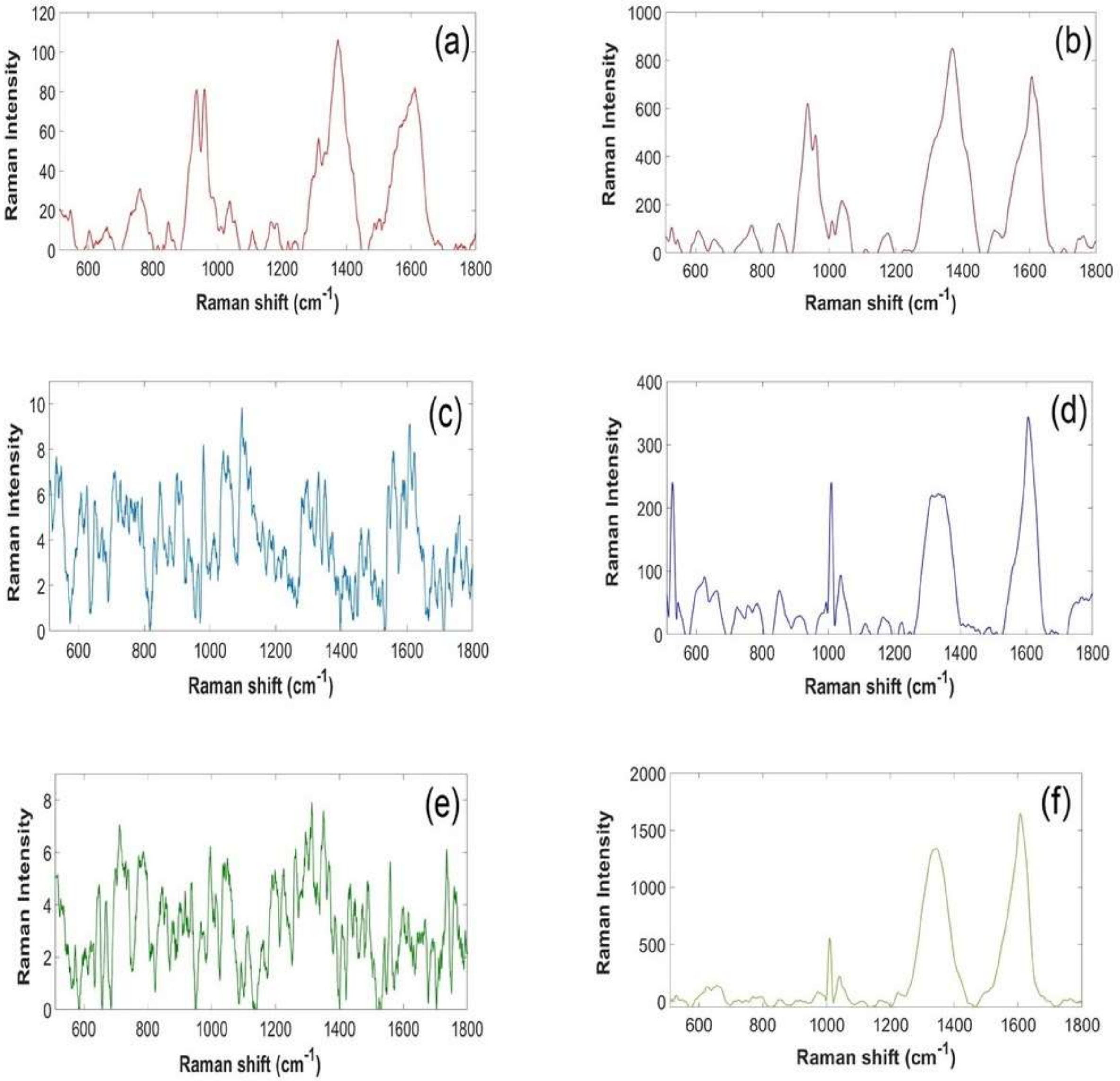

Figure S2: Raman spectra of graphene-coatings: (a and b) GO (N=25) on a flat substrate and Ag dendrites, respectively; (c and d) rGO (N=5) on a flat substrate and Ag dendrites, respectively; (e, f) GNP (N=1) on a flat substrate and Ag dendrites, respectively. A Au-coated (CVD) polished Si wafer was used as flat substrate.

Table S1: Standard deviations and relative standard deviations (%) for the data displayed in Figure 7.

	Standard Deviation (Relative Standard Deviation, %)		
**Spray Nozzle passes (N)**	**GO**	**rGO**	**GNP**
1	0.11 (18%)	0.21 (22%)	0.45 (25%)
5	0.75(55%)	0.53 (21%)	0.50 (52%)
10	0.54 (48%)	1.07 (69%)	0.54 (55%)
15	0.61 (50%)	0. 85 (48%)	0.10 (20%)
20	0.44 (21%)	0.46 (27%)	0.06 (14%)
25	0.64 (27%)	0.20 (22%)	0.23 (33%)
